# Substituent-Adjusted Electrochromic Behavior of Symmetric Viologens

**DOI:** 10.3390/ma14071702

**Published:** 2021-03-30

**Authors:** Qun Zhang, Li Yuan, Fanglan Guan, Xin Li, Rui Wang, Jian Xu, Yanyan Qin, Guangming Chen

**Affiliations:** 1 Beijing Key Laboratory of Clothing Materials R&D and Assessment, Beijing Engineering Research Center of Textile Nanofiber, School of Materials design & Engineering, Beijing Institute of Fashion Technology, Beijing 100029, China; 20180023@bift.edu.cn (Q.Z.); yuanli772716363@126.com (L.Y.); clygfl@bift.edu.cn (F.G.); clywangrui@bift.edu.cn (R.W.); 2 College of Chemical and Environmental Engineering, Low-dimensional Materials Genome Initiative, Shenzhen University, Shenzhen 518055, China; jxu@iccas.ac.cn; 3 College of Materials Science and Engineering, Shenzhen University, Shenzhen 518055, China; qinyanyan@szu.edu.cn

**Keywords:** 4,4′-bipyridine, symmetric viologens, electrochromic, substituent-adjusted

## Abstract

As a promising electrochromic material, viologens have attracted increasing attention due to their high redox activity and adjustable electrochromic capability. In order to investigate the effect of alkyl substituents on electrochromic behavior, four alkyl-substituted viologens and a benzyl-substituted viologen were synthesized, namely 1,1′-dioctyl-4,4′-bipyridinium dibromide (OV), 1,1′-didekyl-4,4′-bipyridinium dibromide (DeV), 1,1′-didodecyl-4,4′-bipyridinium dibromide (DoV), 1,1′-dihexadecyl-4,4′-bipyridinium dibromide (HV), and 1,1′-dibenzyl-4,4′-bipyridinium dibromide (BV). The different photophysical and electrochemical properties of these viologens were attributed to their deviation in spatial structure caused by different substituents. Compared with benzyl-substituted BV, a slight blueshift occurred for the absorption peaks of alkyl-substituted viologens from 262 to 257 nm with the increase in alkyl chain length. Moreover, the first redox couple increased positively, and the dimerization of the compound decreased gradually, accompanied by the decrease in optical contrast and distinct chromatic difference. A comparison of chromatic and optical contrasts indicated that OV had the longest coloring response time (R_Tc_), while it was shortest for HV. The bleaching response time (R_Tb_) of viologen films gradually decreased with the alkyl chain length, and the OV film had the shortest R_Tb_. Furthermore, when increasing the length of the alkyl chain, the cycling stabilities of alkyl viologens increased gradually. In addition, the OV film exhibited the best contrast after 200 continuous cycles.

## 1. Introduction

Electrochromic (EC) materials have attracted growing interest for researchers due to their extensive applications in displays [1,2,3], smart windows [4,5,6,7,8], anti-dazzling mirrors [9,10,11], and optical sensors [12,13,14]. By applying certain voltages, the optical properties of these materials can be reversibly regulated in the visible and the near-infrared regions. Nowadays, research on electrochromic materials has focused on inorganic components such as traditional metal oxides [15,16,17,18,19,20]. Although different strategies have recently been published to increase the coloring efficiency and duration [21,22], inorganic electrochromic materials still have the challenges of slow switching time and monotonous color. Organic electrochromic materials [23,24,25,26,27,28,29,30] have also attracted great interest due to their excellent properties, such as easy structure modification, rich redox states, wide voltage window, and high energy density.

Viologen-based electrochromic materials, including small molecules, polymers, and composite materials, have been extensively studied [31,32,33,34,35,36,37]. As a type of quaternary pyridinium salt, the conjugated bi-pyridyl groups possess excellent electrochromic behavior [38]. Viologens can undergo two steps of reversible reductions that exhibit three redox states [39,40], namely, di-cation (V^2+^), radical-cation (V^+^**^·^**), and neutral species (V^0^) units. Divalent cation V^2+^ is the most stable form and is colorless, while V^0^ is shallow yellow. Radical cations V^+^^·^ are intensely colored with high molar absorption coefficients, owing to the charge transfer between the +1 and 0 valent nitrogen. Moon’s study [41] showed a significantly different EC behavior between monoheptyl viologen (MHV^+^) and diheptyl viologen (DHV^2+^) in flexible electrochromic devices despite having similar chemical structures. The color and spectral characteristics of small molecular viologens can be modulated by structures such as the substituents on the nitrogen atoms. However, the correlation between the substituent structures and EC characterization of viologens has not been systematically investigated.

Herein, four alkyl viologens have been designed and synthesized, namely 1,1′-dioctyl- 4,4′-bipyridinium dibromide (OV), 1,1′-didecyl-4,4′-bipyridinium dibromide (DeV), 1,1′-didohexadecyl-4,4′-bipyridinium dibromide (DoV), and 1′-dihexadecyl-4,4′-bipyridinium dibromide (HV). The effect of the alkyl chain on their electrochromic behaviors has been studied. For comparison, a benzyl viologen, 1,1′-dibenzyl-4,4′-bipyridinium dibromide (BV), was also synthesized to obtain further insight into the different influences between alkyl and aromatic substituents. The photophysical, electrochemical, spectroelectrochemical, and EC properties of the viologens have also been systematically investigated.

## 2. Experimental

### 2.1. Materials and Reagents

4,4′-bipyridine, 1-bromooctane, 1-bromodecane, 1-bromododecane, 1-bromohexadecane, and benzyl bromide were purchased from J&K Scientific Ltd (Beijing, China). Potassium chloride, DMF, acetonitrile, and ethanol were provided by Beijing Chemical Plant (Beijing, China). All reagents were of analytical grade and were used without further purification. The water used in the experiments was deionized.

### 2.2. Synthesis Procedure and Structure Characterizations

**Scheme 1:**
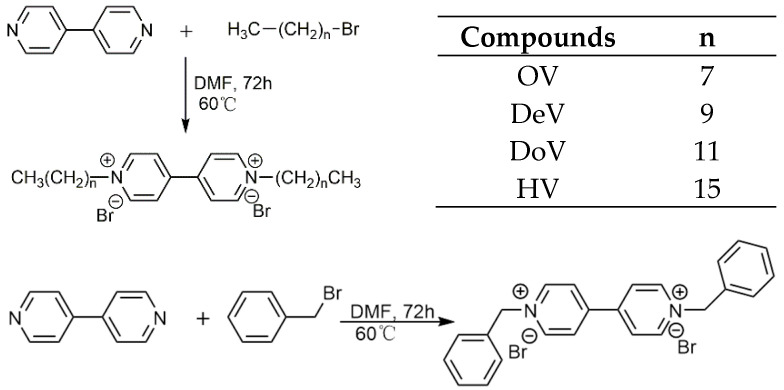
Synthesis route of the viologen compounds with different alkyl chains (n = 7, 9, 11, 15).

Scheme 1 showed the preparation route of substituted viologens. The pyridine precursors with benzyl and different lengths of alkyl groups were synthesized by coupling reactions. The obtained viologen derivatives were characterized by ^1^H NMR (Brucker AVANCE II-500, Brucker, Germany) and FT-IR (Nicolet Nexus 670, Thermo Nicolet Corporation, Madison, WI, USA), and were found to be consistent with the proposed structures.

### 2.3. Synthesis of 1,1′-Dioctyl-4,4′-Bipyridinium Dibromide (OV)

4,4’-bipyridine (1 mmol) and 1-bromooctane (4 mmol) were added into a 100 mL round-bottomed flask. Under a nitrogen atmosphere, 50 mL of DMF was injected into the flask. The reaction system was stirred at 60 °C for 72 h. After cooling to room temperature, the residue was filtered and washed with overdose ether, and it was then recrystallized in acetonitrile to give pure 1,1′-dioctyl-4,4′-bipyridinium dibromides (OV) in a 73% yield with a yellowish brown color. ^1^H NMR (500 MHz, CH_3_OD) (ppm): 9.31 (d, 4H), 8.70(d, 4H), 4.76(t, 4H), 2.12 (d, 4H), 1.25-1.51 (m, 20H), 0.92 (t, 6H). IR (KBr): *v* 2918 cm^−1^, 2841 cm^−1^, 1663 cm^−1^, 1465 cm^−1^, 1220 cm^−1^, 719 cm^−1^.

### 2.4. Synthesis of 1,1′-Didecyl-4,4′-Bipyridinium Dibromide (DeV)

Similarly to the procedure of OV, DeV was synthesized from 1-bromodecane, and a yellow substance in 75% yield was obtained. ^1^H NMR (500 MHz, CH_3_OD) (ppm): 9.30 (d, 4H), 8.70 (d, 4H), 4.75 (t, 4H), 2.11 (d, 4H), 1.25-1.51 (m, 28H), 0.92 (t, 6H). IR (KBr): *v* 2918 cm^−1^, 2841 cm^−1^, 1663 cm^−1^, 1465 cm^−1^, 1224 cm^−1^, 719 cm^−1^.

### 2.5. Synthesis of 1,1′-Didodecyl-4,4′-Bipyridinium Dibromide (DoV)

Similarly to the procedure of OV, DoV was synthesized from 1-bromododecane in a 77% yield with a yellow color. ^1^H NMR (500 MHz, CH_3_OD) (ppm): 9.31 (d, 4H), 8.70 (d, 4H), 4.75 (t, 4H), 2.12 (d, 4H), 1.25–1.51 (m, 36H), 0.92 (t, 6H).IR (KBr): *v* 2919 cm^−1^, 2840 cm^−1^, 1663 cm^−1^, 1465 cm^−1^, 1224 cm^−1^, 719 cm^−1^. 

### 2.6. Synthesis of 1,1′-Hexadecyl-4,4′-Bipyridinium Dibromide (HV)

Similarly to the procedure of OV, HV with the yellow color was synthesized from 1-bromohexadecane in a 79% yield. ^1^H NMR (500 MHz, CH_3_OD) (ppm): 9.30 (d, 4H), 8.70(d, 4H), 4.78 (t, 4H), 2.10 (d, 4H), 1.25-1.50 (m, 52H), 0.91 (t, 6H). IR (KBr): *v* 2919 cm^−1^, 2841 cm^−1^, 1663 cm^−1^, 1465 cm^−1^, 1221 cm^−1^, 719 cm^−1^.

### 2.7. Synthesis of 1,1′-Dibenzyl-4,4′-Bipyridinium Dibromide (BV)

Similarly to the procedure of OV, BV with a bright yellow color was synthesized from benzyl bromide in a 77% yield. ^1^H NMR (500 MHz, CH_3_OD) (ppm): 9.30 (d, 4H), 8.70 (d, 4H), 7.61 (m, 4H), 7.52 (m, 6H), 4.78 (t, 4H). IR (KBr): *v* 2991 cm^−1^, 2941 cm^−1^, 2841 cm^−1^, 1663 cm^−1^, 1443 cm^−1^, 1219 cm^−1^, 743 cm^−1^, 696 cm^−1^.

### 2.8. Performance Measurements

Photophysical measurements were recorded on a Perkin Elmer Lambda 750 UV-Visible spectrophotometer (Perkin Elmer, Akron, OH, America). The redox behavior of the viologens were examined with cyclic voltammetry (CV), which was performed with an electrochemical testing system (Zahner IM6, Zahner, Germany) at a sweep rate of 50 mV/s at voltages from 0 to −1.2 V, and under a square wave step potential from 0 to −0.7 V each for 15 s. The test system was characterized by the three-electrode system in the presence of 0.1 M of KCl solution. Although the pH of KCl is near-neutral, it is a strong electrolyte that has been used in many studies [42]. Platinum wire and saturated calomel were used as the counter and reference, respectively. The viologen-coated indium tin oxide (ITO) glass slide with a viologen film thickness of about 2 μm and an area of ca. 0.8 × 2 cm^2^ was used as the working electrode. The multipotential UV spectra were determined by a UV-visible spectrophotometer linked with a Zahner IM6 workstation. The color difference of the film was analyzed using the SC-80C automatic colorimeter of Beijing Kangguang Instrument Co., Ltd (Beijing, China). All data were directly obtained from the instrument, and the data error was basically the instrument error.

## 3. Results and Discussion

### 3.1. Photophysical Properties

**Figure 1:**
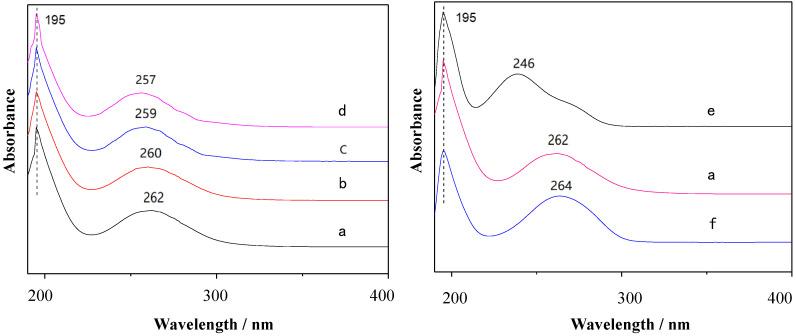
The ultraviolet spectra of 4,4′-pyridine and substituted viologens: (a) OV, (b) DeV, (c) DoV, (d) HV, (e) 4, 4′-pyridine, and (f) BV.

The photophysical properties of the substituted viologens were investigated in 0.05 mmol/L of KCl solution by UV-vis absorption spectroscopy, as shown in Figure 1. All substituted viologens exhibited the same absorption bands at 195 nm, which were attributed to the n→δ* transition absorption of N atoms on the pyridine ring [43]. With the increase in alkyl chain length, the maximum absorption wavelength of four alkyl viologens blueshifted from 262 nm to 257 nm. OV exhibited a maximum absorption wavelength at 262 nm while HV showed a minimum absorption wavelength at 257 nm. These results indicated that the absorption peak of the B band in the π–π* transition of the pyridine ring was slightly blueshifted due to the increase in alkyl chain length, which weakened the effective conjugation because of the alkyl chains’ space effect. On the other hand, the absorption maximum of benzyl viologens was located at 264 nm due to the conjugation of the aromatic ring.

### 3.2. Electrochemical Properties

**Figure 2:**
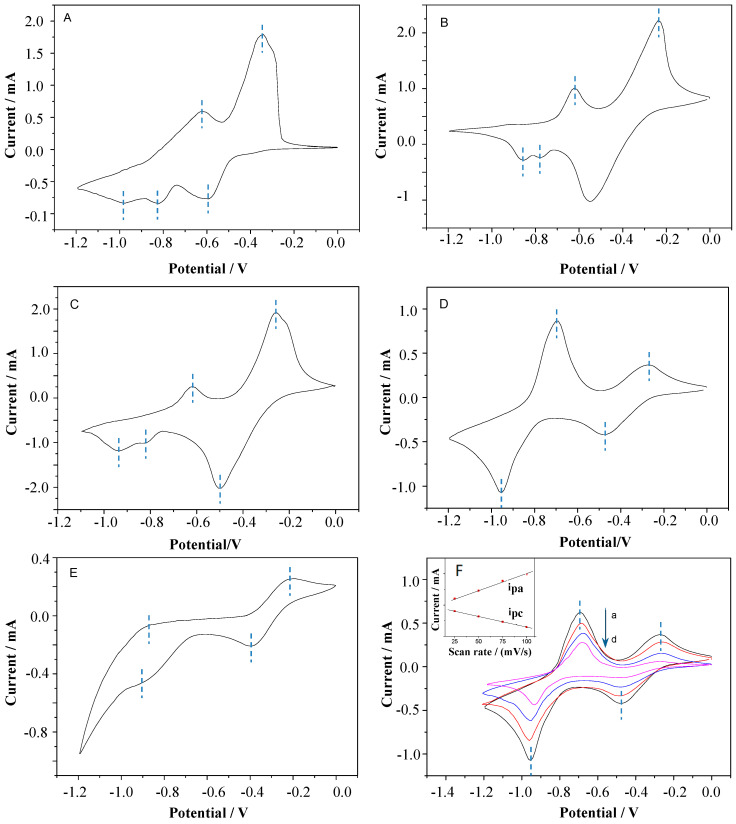
CVs for (**A**) OV, (**B**) DeV, (**C**) DoV, (**D**) HV, and (**E**) BV scanned from 0 to −1.2 V in 0.1 M of KCl solution at a scan rate of 50 mV/s. (**F**) CV of HV films at different scanning velocities (a: 100, b: 75, c: 50, and d: 25 mV/s).

To investigate the electrochemical properties of the target viologens, cyclic voltammetry (CV) measurements were carried out. As shown in Figure 2A–E, these curves are similar, and all substituted viologens underwent reversible two-electron reduction and single electron oxidation [44]. The two couples of redox peaks obtained during the cathode scan can be attributed to the two one-electron redox processes of the 4,4′-bipyridine units, corresponding to the generation of radical cationic and neutral species [45]. The redox potentials of the five viologens are shown in Table 1. As seen from Table 1 and Figure 2A, the OV film exhibited two redox processes, which were the anodic wave potentials of −0.34 and −0.60 V, and the cathodic wave potentials of −0.62 and −0.98 V. The first redox couple of −0.34 and −0.60 V was correlated with the redox reaction between the divalent cation OV^2+^ and radical cation OV^+•^, respectively. The second couple of −0.62 and −0.98 V was assigned to the OV^+•^ and di-reduced species OV^0^, respectively. When the octyl in OV was replaced by a decyl unit, the first redox couple (−0.27 V, −0.55 V) of the HV film was increased (Figure 2B), and the value of the first redox couple continued to grow in a positive direction with the increase in the carbon chain.

As the alkyl chain lengthened, the oxidation potential of the second redox couple shifted negatively while the reduction potential had no obvious change. As shown in Figure 2E and Table 1, due to the aromatic benzyl substituent, the redox potential of BV was obviously different from those of alkyl viologens. The redox wave of the second redox couple was wide and not sharp, and the second oxidation potential was much higher than those of alkyl-substituted viologens, meaning that the reversibility of the second couple of redox peaks worsened a little more.

Otherwise, the oxidation peaks of −0.82 V (Figure 2A,C) and −0.78 V (Figure 2B) were caused by the dimerization of viologens. However, with the increase in carbon chain length, the steric hindrance effect of the substituents increased in the same amount of time, which decreased the dimerization of the compounds, and the oxidation wave of about −0.82 V gradually decreased until it could be ignored (Figure 2D). Figure 2F shows the CVs of the HV film at different scanning potentials. With the increase in scanning rate, the oxidation potential increased while the reduction potential decreased, and the oxidation current and reduction current showed a linear relationship with the scanning rate. These results indicate that the redox events were controlled by the surface-confined electron transfer kinetics rather than the diffusion process [32,33].

### 3.3. EC Behaviors

#### 3.3.1. Multipotential UV Absorption Spectroscopy

**Figure 3:**
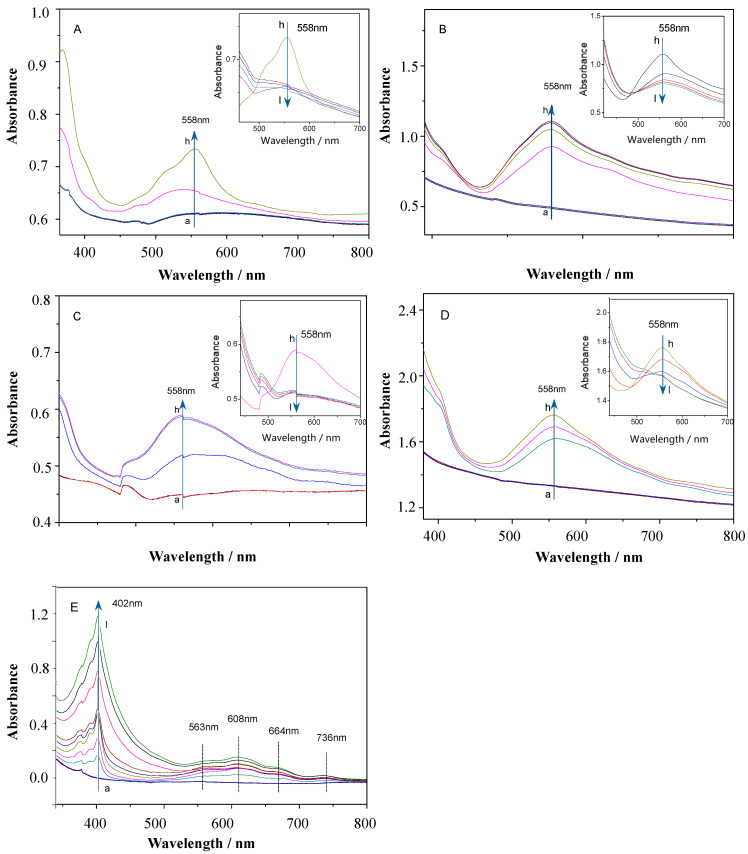
Absorption spectra of the (**A**) OV, (**B**) DeV, (**C**) DoV, (**D**) HV, and (**E**) BV upon applying different potentials. (a: 0, b: −0.1, c: −0.2, d: −0.3, e: −0.4, f: −0.5, g: −0.6, h: −0.7, i: −0.8, j: −0.9, k: −1.0, and l: −1.1 V).

The EC behaviors of the target viologens were further studied by measuring their spectro-electrochemical properties. The absorption spectra of viologens films were obtained with external potentials from 0 to −1.1 V in 0.1 M of KCl as the electrolyte solution. As shown in Figure 3A–D, all the alkyl-substituted viologens exhibited similar absorption spectra. Taking Figure 3A (OV) as an example, when voltages from 0 to −0.5 V were applied, the absorption spectra of the OV film exhibited no changes, indicating no electrochromic reaction taking place. When the potential stepped from −0.5 to −0.7 V, the absorbance band became clearer. Under −0.7 V, the maximum absorbance peak was characterized at 558 nm with the color change into purplish-red. Human eyes are especially sensitive to the wavelength of 558 nm where the largest optical attenuation for the OV film is also obtained. As the potential continued to increase negatively, the strong absorption bands around 558 nm gradually decreased, and the color changed from purplish-red to light yellow. These phenomena also corresponded to the conversion between OV^2+^ and OV^+•^, which indicated the occurrence of the second redox process, and a yellow-brown color of OV^0^ was generated [30].

Absorption spectra of the BV film (Figure 3E) were obviously different from those mentioned above. The reduction of benzyl viologen was produced in the absorption spectra. Applying a negative potential on the BV film, the maximum absorption band was characterized at 402 nm. With the increase in the reductive potentials, new absorption bands around 563, 608, 664, and 736 nm continuously appeared in the visible region, leading to an obvious color change from blue to grayish yellow.

#### 3.3.2. Color Analysis

Detailed color parameters, where L* represents lightness, and a* and b* describe the chromaticity and saturability, respectively, are listed in Table 2. The color of the OV film was revealed to turn from light yellow to purple when the voltage changed from −0.9 to −0.6 V, in which the chromatic difference (ΔEab*) was 61.61. As the length of the alkyl carbon number increased, the color changes were different and the ΔEab* of DeV, DoV, and HV decreased to 45.88, 36.59, and 21.25, respectively. The BV film showed a lower ΔEab* than that of OV but a higher ΔEab* than those of other alkyl viologens, coupled with a color change from grayish yellow to blue due to the aromatic ring.

#### 3.3.3. Contrast and Response time

A square-wave potential step method for sample films was employed to investigate the optical contrast and response time. In this test, 0 and −0.7 V were used as the reduced and oxidized state voltage, respectively, in which each voltage lasted for 15 s.

**Figure 4:**
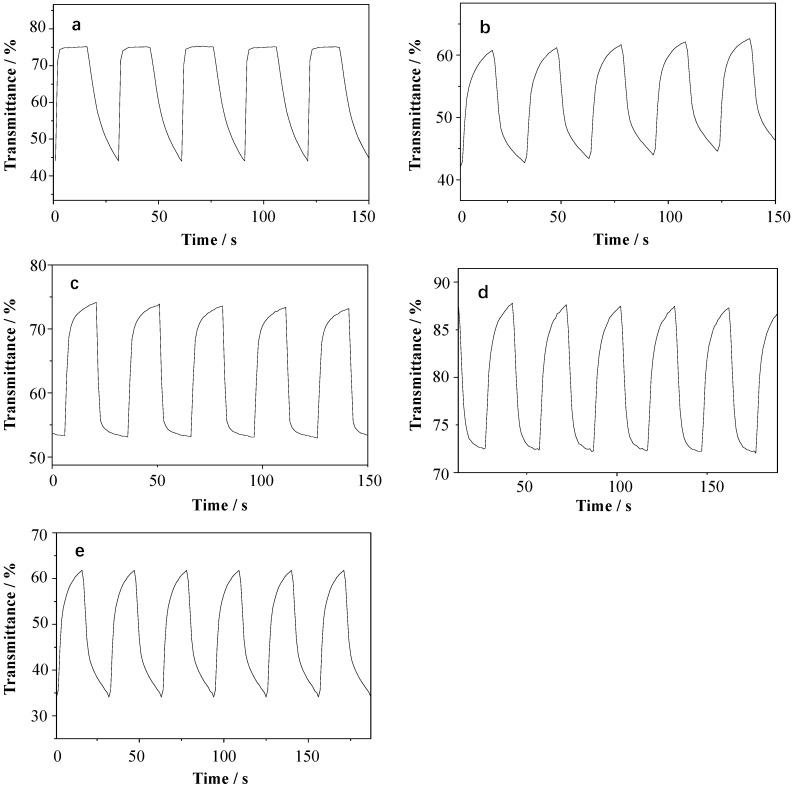
The square wave transmittance curves of different viologen films: (**a**) OV, (**b**) DeV, (**c**) DoV, (**d**) HV, and (**e**) BV, in 0.1 M of KCl solution.

Figure 4 and Table 3 display the graphs and data sheets of the transmittance for the alkyl viologen films at 558 nm a test interval time of 15 s. Figure 4a describes the optical transmittance of the OV film. The coloring response time (R_Tc_) and bleaching response time (R_Tb_) were defined as the time required to reach 90% of the total change in transmittance after switching the potential. The OV film bleached and colored when the voltage changed from 0 V to −0.7 V, obtaining a maximum contrast (ΔT_max_) of 33%. The R_T__b_ and R_Tc_ of the OV film were 0.95 s and 8.16 s (Table 3), respectively.

As shown in Table 3, when increasing the length of the alkyl chain, both ΔT_max_ and R_Tc_ of the alkyl viologens decreased. OV showed the longest R_Tc_ while HV showed the shortest R_Tc_. As the length of the alkyl carbon chain increased, the R_Tb_ of the DeV, DoV, and HV films gradually decreased to 6.72 s, 5.38 s, and 4.25 s, respectively, except the OV film, which had the shortest R_Tb_ of 0.97 s. Both the R_Tb_ and R_Tc_ of the BV film were shorter than those of DeV in these samples, and the ΔT_max_ was only lower than that of OV but better than those of DeV, DoV, and HV.

#### 3.3.4. Cyclic Stability

**Figure 5:**
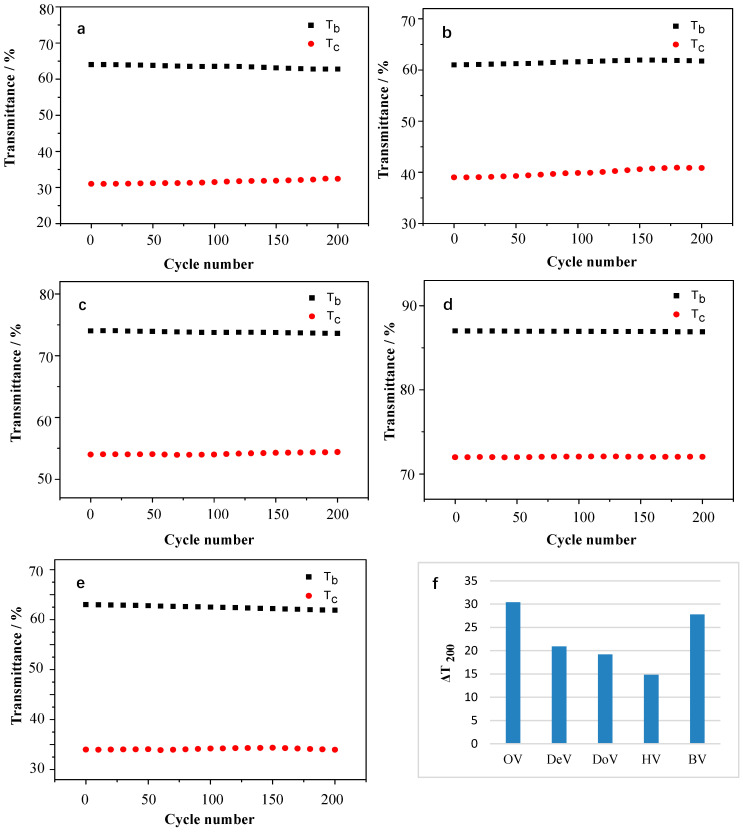
The transmittance (**a**–**e**) and ΔT_200_ (**f**) of OV, DeV, DoV, HV, and BV films under continuous cycling between colored and bleached states.

The cyclic stability is considered an important requirement for further practical applications. Therefore, the stability of the resulting viologen film was investigated by continuously cycling the devices between the colored and the bleached states, and the percentages of the retained transmittance after 200 cycles (ΔT_200_) are given in Figure 5f.

Figure 5a–e displays the transmittance of the viologen film over 200 continuous cycles. As shown in Figure 5f, after 200 cycles, OV still showed the best transmittance contrast. The losses of transmittance contrast values for OV, DeV, DoV, and HV were 7.9%, 4.5%, 4.0%, and 1.3%, respectively, indicating that the resulting viologens films became more stable with the increase in the alkyl chain length in the visible region for the EC applications. OV still showed the best optical contrast, but HV showed the most stability in continuous cycles due to the long carbon chain, which can minimize the effect of dimerization. The ΔT_200_ of BV was lower than that of OV, but better than those of the others.

## 4. Conclusions

In this work, four alkyl-substituted and benzyl-substituted viologens have been designed and successfully synthesized. The influence of the different structures of the substituents on the electrochromism properties has been investigated. Although they all showed a rapid response time, good optical contrast, and stable long-time operation, the difference between the substituent structures reflected different capability trends. UV-vis absorption spectroscopy showed that the absorption peak of the B band in the π-π* transition of the pyridine ring was slightly blueshifted due to the increase in the alkyl chain length, while the absorption maximum of benzyl viologens was located at 264 nm. The CV curves confirmed that the first redox couple was increased in a positive direction with the increase in alkyl chain length. In addition, the R_Tc_ of the alkyl substituent viologens was improved while the optical contrasts decreased gradually with the increase in the alkyl chain length. The OV film showed the maximum contrast even after 200 cycles, but the HV film showed the most stability because of the minimum dimerization. The BV film showed a better chromatic difference and ΔT_max_ than those of DeV, DoV, and HV, but a lower performance than that of OV.

## Figures and Tables

**Table 1:** Cyclic voltammograms data of substituent viologens.

Substituent Viologens	First Oxidation Potential Ep_a1_(V)	Second Oxidation Potential Ep_a2_(V)	First Reduction Potential Ep_c1_(V)	Second Reduction Potential Ep_c2_(V)
OV	−0.34	−0.60	−0.62	−0.98
DeV	−0.27	−0.62	−0.55	−0.86
DoV	−0.24	−0.62	−0.50	−0.94
HV	−0.24	−0.70	−0.47	−0.95
BV	−0.21	−0.87	−0.39	−0.90

**Table 2:** Color variations and chroma of viologen films under different potentials.

Viologen Films	Color Variations	Potential/V	L *	a *	b *	ΔEab *
OV	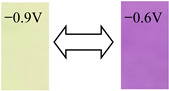	−0.9 −0.6	62.17 69.38	−6.43 35.22	33.12 −11.70	61.61
DeV	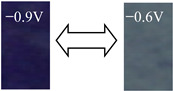	−0.9 −0.6	53.37 56.27	31.52 3.69	−49.34 −12.67	45.88
DoV	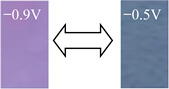	−0.9 −0.5	56.38 61.25	20.45 −2.86	−4.36 −32.14	36.59
HV	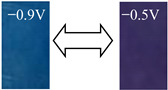	−0.9 −0.5	59.34 63.70	2.58 23.15	−41.21 −43.47	21.25
BV	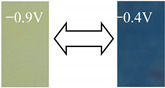	−0.9 −0.4	48.86 54.37	−12.53 9.35	10.68 −37.76	53.44

**Table 3:** Electrochromic (EC) switching properties of vilogen films.

Viologen Films	R_Tb_ (s)	R_Tc_ (s)	ΔT_max_ (%)
OV	0.95	8.16	33
DeV	6.72	5.42	22
DoV	5.38	4.35	20
HV	4.25	3.23	15
BV	5.94	4.56	29

## Data Availability

The data presented in this study are available on request from the corresponding author.
